# New-onset COVID-19–related diabetes: an early indicator of multi-organ injury and mortally of SARS-CoV-2 infection

**DOI:** 10.1007/s44194-022-00006-x

**Published:** 2022-05-26

**Authors:** Jin-Kui Yang, Miao-Miao Zhao, Jian-Min Jin, Shi Liu, Peng Bai, Wei He, Fei Wu, Xiao-Fang Liu, Zhong-Lin Chai, De-Min Han

**Affiliations:** 1 Department of Endocrinology, Beijing Tongren Hospital, Capital Medical University, Beijing, 100730 China; 2 Department of Respiratory and Critical Care Medicine, Beijing Tongren Hospital, Capital Medical University, Beijing, 100730 China; 3 Department of Internal Medicine, Union Hospital, Tongji Medical College, Huazhong University of Science and Technology, Wuhan, 430022 China; 4 Department of Critical Care Medicine, Beijing Tongren Hospital, Capital Medical University, Beijing, 100730 China; 5 Department of Diabetes, Central Clinical School, Monash University, Melbourne, Australia; 6 Department of Otolaryngology and Head Surgery, Beijing Tongren Hospital, Capital Medical University, Beijing, 100730 China

**Keywords:** COVID-19, SARS-CoV-2, Blood glucose, Multi-organ injury, Mortality, Predictor

## Abstract

**Objective:**

The pandemic of 2019 coronavirus (SARS-CoV-2) disease (COVID-19) has imposed a severe public health burden worldwide. Most patients with COVID-19 were mild. Severe patients progressed rapidly to critical condition including acute respiratory distress syndrome (ARDS), multi-organ failure and even death. This study aims to find early multi-organ injury indicators and blood glucose for predicting mortality of COVID-19.

**Methods:**

Fasting blood glucose (FBG) ≥7.0 mmol/L for two times during hospitalization and without a history of diabetes were defined as new-onset COVID-19-related diabetes (CRD). Indicators of injuries for multiple organs, including the lung, heart, kidney and liver, and glucose homeostasis were specifically analyzed for predicting death.

**Results:**

A total of 120 patients with a severity equal to or greater than *Moderate* were hospitalized. After excluding patients with history of diabetes, chronic heart, kidney, and liver disease, 69 patients were included in the final analysis. Of the 69 patients, 23 were *Moderate*, 20 were *Severe*, and 26 were *Critical* (including 16 deceased patients). Univariable analysis indicated that CRD, lactate dehydrogenase (LDH), hydroxybutyrate dehydrogenase (HBDH), creatine kinase (CK) and creatinine (Cr) were associated with death. Multivariable analysis indicated that CRD was an independent predictor for death (HR = 3.75, 95% CI 1.26–11.15). Abnormal glucose homeostasis or CRD occurred earlier than other indicators for predicting poor outcomes. Indicators of multiple organ injury were in parallel with the expression patterns of ACE2 (the SARS-CoV-2 receptor) in different organs including pancreatic islet.

**Conclusions:**

New-onset COVID-19-related diabetes is an early indicator of multi-organ injury and predictor for poor outcomes and death in COVID-19 patients. As it is easy to perform for clinical practices and self-monitoring, glucose testing will be helpful for predicting poor outcomes to facilitate appropriate intensive care.

## Background

In early December 2019, an outbreak of a novel coronavirus (SARS-CoV-2) pneumonia disease (COVID-19) occurred in Wuhan, China (Zhu et al. 2020). Most patients with COVID-19 were *Mild*. *Moderate* patients often experienced dyspnea after 1 week. Some *Severe* patients progressed rapidly to *Critical* condition including multi-organ failure and even death (Huang et al. 2020; Chen et al. 2020; Cao et al. 2020; Xu et al. 2020a).

COVID-19 is caused by severe acute respiratory syndrome coronavirus 2 (SARS-CoV-2) infection. It is reminiscent of the SARS-CoV outbreak in early 2003, because both coronavirus attack cells via the same receptor, angiotensin-converting enzyme 2 (ACE2) (Zhu et al. 2020). As early as 2006, for the first time, we reported that acute diabetes was commonly present in SARS patients without prior history of diabetes and without using glucocorticoids, and was an independent predictor for mortality in SARS patients (Yang et al. 2006). Acute diabetes was commonly present in SARS patients without prior history of diabetes and without using glucocorticoids, and was an independent predictor for mortality in SARS patients (Yang et al. 2010).

ACE2 are expressed in key metabolic organs and tissues, including pancreatic islet cells (Fang and Yang 2010) the small intestine, adipose tissue, and the kidneys (Hamming et al. 2004). Thus, it is plausible that COVID-19 may have pleiotropic alterations of glucose metabolism or new type of disease (Rubino et al. 2020). Recently, new-onset diabetes in COVID-19 is noticed. An international group of leading diabetes researchers participating in the CoviDIAB Project have established a global registry of patients with Covid-19–related diabetes (covidiab.e-dendrite.com) (Rubino et al. 2020). In this observational cohort study, we aim to explore the correlation between COVID-19–related diabetes (CRD) and poor outcomes or death in patients with COVID-19.

## Methods

### Participants

This retrospective cohort study included consecutive COVID-19 adult inpatients (≥18 years old) admitted to Wuhan Union Hospital (Wuhan, China) and treated by the supportive medical team of Beijing Tongren Hospital (Beijing, China) from January 29, 2020, to March 20, 2020.

Respiratory specimens were collected by the local center for disease control and prevention (CDC) and then shipped to designated authoritative laboratories to detect SARS-CoV-2. The presence of SARS-CoV-2 in respiratory specimens was detected by real-time RT-PCR methods. The RT-PCR assay was conducted as per the protocol established by the World Health Organization (WHO).

#### Exclusion criteria

(1) Patients with a history of type 1 or type 2 diabetes were excluded. (2) To avoid glucocorticoid-induced diabetes, patients who received glucocorticoid treatment were excluded. (3) For the identification of indicators of early multi-organ injury for predicting poor outcomes, patients with a history of heart disease (myocardial infarction and heart failure), kidney disease (maintenance dialysis or renal transplantation), or liver disease (liver cirrhosis) were excluded.

#### CRD

FBG ≥ 7.0 mmol/L for two times during hospitalization, without glucocorticoid treatment, and without a history of diabetes in COVID-19 patients were defined as CRD.

### Clinical classification of COVID-19

Diagnosis and clinical classification criteria and treatment plan (version 6.0) of COVID-19 was launched by the National Health Committee of China (http://www.nhc.gov.cn/). The clinical classification of severity is as follows: (1) *Mild*, having only mild symptoms, imaging shows no pneumonia. (2) *Moderate*, with fever, respiratory tract symptoms, and imaging shows pneumonia. (3) *Severe*, meet any of the following signs: a) respiratory distress, respiratory rate ≥ 30 beats / min; b) in the resting state, finger oxygen saturation ≤ 93%) arterial blood oxygen partial pressure (PaO_2_/oxygen concentration (FiO_2_) ≤ 300 mmHg (1 mmHg = 0.133 kPa). (4) *Critical*, one of the following conditions: a) respiratory failure occurs and requires mechanical ventilation; b) Shock occurs; c) ICU admission is required for combined organ failure.

### Data collection

Demographic, clinical, laboratory and outcome data were extracted from the electronic hospital information system using a standardized form. All medical data were checked by two medical doctors (JMJ and PB) and the leader author (JKY) adjudicated any different interpretation between the two medical doctors.

### Immunohistochemical patterns of ACE2

Lung, pancreas, heart, kidney and liver tissue was obtained from a brain-dead organ donor after informed consent from his wife. The protocol was approved by the ethics committee of Beijing Tongren Hospital, Capital Medical University. Serial sections were made from each of the tissues, and ACE2 immunohistochemical staining was compared with negative controls. A qualified pathologist interrelated the results of ACE2 expression.

### Statistical analysis

Data were expressed as median [interquartile range (IQR)] or percentage, as appropriate. Comparison of continuous data among different severity groups and between the deceased and survivor groups were determined using a Kruskal-Wallis test and Mann-Whitney U test, respectively. Chi-square (χ^2^) test for trend and two-group Chi-square tests or Fisher’s exact tests were used for categorical variables as appropriate. Proximity Matrix and Dendrogram of six measures as different organ damage indicators were calculated and developed by Hierarchical cluster analysis. To find the risk factors predicting in-hospital death, univariate and multivariate Cox proportional hazard model were used to calculated hazard ratio (HR). Kaplan–Meier survival curves and the log-rank test were used for testing the survival between normal and abnormal indicators. SPSS for Windows 17.0 and Graphpad prism 7.0 software were used for statistical analysis, with statistical significance set at 2-sided *P* < 0.05.

## Results

### Demographic and clinical characteristics of the COVID-19 adult inpatients

A total of 120 consecutive COVID-19 adult inpatients (≥18 years old) were admitted to Wuhan Union Hospital (Wuhan, China) and treated by the supportive medical team of Beijing Tongren Hospital (Beijing, China) from January 29, 2020 to March 20, 2020. Because Wuhan Union Hospital was assigned responsibility for the treatments of severe COVID-19 patients by the Wuhan government, most patients were *Severe* or *Critical*, some were *Moderate*, but none were *Mild*.

After excluding patients received glucocorticoid treatment or with a history of diabetes, myocardial infarction, heart failure, dialysis, renal transplant, cirrhosis, and patients missing basic medical information, 69 patients were included in the final analysis. Of the 69 patients, 23 were *Moderate*, 20 were *Severe*, and 26 were *Critical* (including 16 deceased patients). The median age of the 69 patients was 61 years (IQR, 52 to 67). Older patients tend to be more serious (*P* = 0.024). The ratio of male to female patients were relatively similar, with 49.3% males. However, the proportion of males in deceased patients were 81.2%, suggestive that males may have higher mortality from COVID-19 (*P* = 0.003) as we previously and firstly reported (Jin et al. 2020). Fever (89.9), cough (65.2%) and dyspnea (43.5%) were the most common symptoms, while sputum (17.4%) and diarrhea (17.4%) were less common. An increase in white blood cells and neutrophils, and decrease in lymphocytes and platelets occurred in *Critical* patients at the time of admission. A decrease of albumin, an increase of globulin, and an increase of C-reactive protein was also present in *Critical* patients (Table 1).

**Table 1:** Demography, clinical and laboratory parameters of patients with COVID-19

	All patients (*n* = 69)	Severity			
		Moderate (*n* = 23)	Severe (*n* = 20)	Critical (*n* = 26)	*P* value
Age—years	61 (52–67)	56 (47–64)	62 (55–67)	65 (57–71)	**0.024**‡
Male sex—n (%)	34 (49.3)	9 (39.1)	8 (40.0)	16 (61.5)	0.111#
Symptoms					
Fever—n (%)	62 (89.9)	18 (78.3)	19 (95.0)	25 (96.2)	**0.041**#
Cough—n (%)	45 (65.2)	13 (56.5)	13 (65.0)	19 (73.1)	0.225#
Sputum—n (%)	12 (17.4)	5 (21.7)	4 (20.0)	3 (11.5)	0.341#
Dyspnea—n (%)	30 (43.5)	7 (30.4)	7 (35.0)	16 (61.5)	**0.026**#
Fatigue—n (%)	26 (37.7)	8 (34.8)	6 (30.0)	12 (46.2)	0.397#
Diarrhea—n (%)	12 (17.4)	2 (8.7)	5 (25.0)	5 (19.2)	0.350#
From onset to hospitalization—days	12.0 (9.0–14.0)	13.0 (10.0,15.0)	11.5 (7.8,14.0)	12.0 (7.0,14.0)	0.273‡
Haemoglobin—g/L	127 (116–139)	124 (117–137)	119 (110–143)	130 (121–147)	.477‡
Platelet count— × 10^9^/L	211(183–282)	215 (192–286)	215 (196–293)	205 (159–263)	.515‡
White blood cell count— × 10^9^/L	6.2 (4.7–8.2)	5.7 (4.3–7.4)	5. 9 (4.8–8.1)	7.7 (5.9–9.7)	**.009**‡
Neutrophil count— × 10^9^/L	4.5 (3.1–6.7)	3.3 (2.7–4.9)	4.2 (3.7–5.9)	6.7 (5.4–8.8)	.**000**‡
Lymphocyte count— × 10^9^/L	1.0 (0.7–1.4)	1.3 (0.9–1.7)	0.9 (0.7–1.4)	0.7 (0.4–1.1)	**.000**‡
Potassium—mmol/L	3.9 (3.5–4.3)	4.1 (3.9–4.4)	3.8 (3.5–4.5)	3.7 (3.2–4.0)	**.025**‡
Sodium—mmol/L	139 (137–142)	140 (138–142)	141 (138–143)	138 (135–141)	.078‡
Chloride—mmol/L	104 (99–106)	105 (102–106)	104 (100–108)	103 (98–105)	.109‡
Albumin—g/L	29 (27–34)	34 (30–39)	28 (26–31)	28 (25–30)	**.001**‡
Globulin—g/L	32 (29–36)	30 (26–34)	32 (30–38)	34 (30–39)	**.027**‡
C-reactive protein—mg/L	36 (5–77)	4 (1–36)	23 (7–65)	60 (47–100)	**.000**‡
Alanine aminotransferase—U/L	38 (24–52)	39 (21–49)	37 (24–55)	36 (27–52)	.641‡
Aspartate aminotransferase—U/L	33 (23–47)	23 (17–34)	35 (28–50)	35 (29–56)	**.007**‡
Alkaline phosphatase—U/L	57 (48–67)	55 (46–70)	55 (41–62)	61 (53–79)	.067‡
Lactate dehydrogenase—U/L	312 (204–483)	196 (137–251)	294 (242–384)	499 (354–587)	**.000**‡
Hydroxybutyrate dehydrogenase—U/L	253 (190–368)	161 (118–218)	252 (192–329)	387 (313–478)	**.000**‡
Creatine kinase—U/L	61 (47–143)	48 (36–66)	65 (41–122)	107 (54–275)	**.007**‡
Creatine kinase–MB—U/L	12 (9–19)	9 (7–12)	13 (9–21)	14 (10–23)	**.006**‡
Blood urea nitrogen—mmol/L	5.0 (3.4–6.7)	4.2 (3.3–5.5)	4.5 (2.9–6.9)	5.4 (4.0–7.7)	.158‡
Creatinine—μmol/L	66 (53–84)	64 (52–70)	61 (51–92)	70 (58–93)	.121‡
Fasting Blood Glucose—mmol/L	6.5 (5.7–7.6)	5.7 (5.3–6.5)	6.3 (5.6–6.8)	7.5 (6.4–8.7)	**.000**‡

Data are median (IQR) or n (%). *P* values were calculated by Kruskal-Wallis Test (‡) or χ^2^ test (#), as appropriate for group comparison analyses

### Clinical characteristics of the patients with new-onset CRD

According to the definition of CRD, of the 69 patients, 21 and 48 were defined with CRD and non-CRD respectively. The median age and the ratio of male to female were comparable between CRD and non-CRD groups. The most common symptoms at the time of admission including fever, sputum, dyspnea, fatigue, and diarrhea were comparable, except cough (85.7% vs. 56.3%, *P* = 0.037). An increase in neutrophils occurred in CRD patients at the time of admission. A decrease of sodium, chloride, and globulin, and an increase of alanine aminotransferase (ALT), Aspartate aminotransferase (AST), Alkaline phosphatase (AKP), Lactate dehydrogenase (LDH), hydroxybutyrate dehydrogenase (HBDH), creatine kinase (CK), and CK-MB was also present in CRD patients. Most importantly, the death rate was much higher in CRD group than in non-CRD group (42.9% vs. 14.6, *P* = 0.000) (Table 2).

**Table 2:** Comparation of clinical features between patients with and without new-onset COVID-19–related diabetes (CRD)

	Non-CRD *n* = 48	CRD *n* = 21	***P***
Age—years	61 (52–67)	63 (57–71)	0.435
Male sex—n (%)	20 (41.7)	14 (66.7)	0.056#
Symptoms			
Fever—n (%)	42 (87.5)	20 (95.2)	0.585#
Cough—n (%)	27 (56.3)	18 (85.7)	**0.037**#
Sputum—n (%)	9 (18.8)	3 (14.3)	0.916#
Dyspnea—n (%)	20 (41.7)	10 (47.6)	0.646#
Fatigue—n (%)	19 (39.6)	7 (33.3)	0.622#
Diarrhea—n (%)	11 (22.9)	1 (0.5)	0.121#
From onset to hospitalization—days	13 (10–14)	12 (7–14)	0.564†
Haemoglobin—g/L	123 (115–135)	134 (121–148)	0.067
Platelet count— × 10^9^/L	211 (1191–292)	205 (162–252)	0.483
White blood cell count— × 10^9^/L	6.1 (4.6–8.1)	7.5 (5.6–8.2)	0.062
Neutrophil count— × 10^9^/L	4.0 (3.0–6.0)	6.0 (4.1–7.1)	**0.026**
Lymphocyte count— × 10^9^/L	1.0 (0.7–1.4)	0.9 (0.4–1.3)	0.105
Potassium—mmol/L	3.9 (3.5–4.4)	3.8 (3.5–4.1)	0.962
Sodium—mmol/L	140 (138–142)	136 (135–140)	**0.002**
Chloride—mmol/L	104 (101–107)	101 (98–104)	**0.017**
Albumin—g/L	30 (27–35)	28 (25–30)	0.095
Globulin—g/L	34 (30–40)	32 (28–36)	**0.014**
C-reactive protein—mg/L	15 (8–41)	50 (23–72)	0.073†
Alanine aminotransferase—U/L	31 (23–48)	48 (31–59)	**0.007**
Aspartate aminotransferase—U/L	30 (20–40)	36 (29–64)	**0.015**
Alkaline phosphatase—U/L	54 (47–64)	60 (57–73)	**0.017**
Lactate dehydrogenase—U/L	312 (204–483)	432 (263–547)	**0.006**
Hydroxybutyrate dehydrogenase—U/L	218 (156–298)	368 (303–461)	**0.001**
Creatine kinase—U/L	54 (40–94)	111 (62–267)	**0.010**†
Creatine kinase–MB—U/L	10 (8–13)	16 (10–22)	**0.037**†
Blood urea nitrogen—mmol/L	4.3 (3.4–6.3)	5.9 (4.0–8.0)	0.763
Creatinine—μmol/L	65 (52–74)	76 (58–93)	0.901
Fasting Blood Glucose—mmol/L	5.9 (5.4–6.5)	8.1 (7.6–10.9)	**0.000**
Deceased—n (%)	7 (14.6)	9 (42.9)	**0.000#**

Data are median (IQR) or n (%). P values were calculated by student *t* test or Mann-Whitney U Test (†) or χ^2^ test (#), as appropriate for group comparison analyses

### ACE2 expression and multi-organs injury indicators

**Fig. 1:**
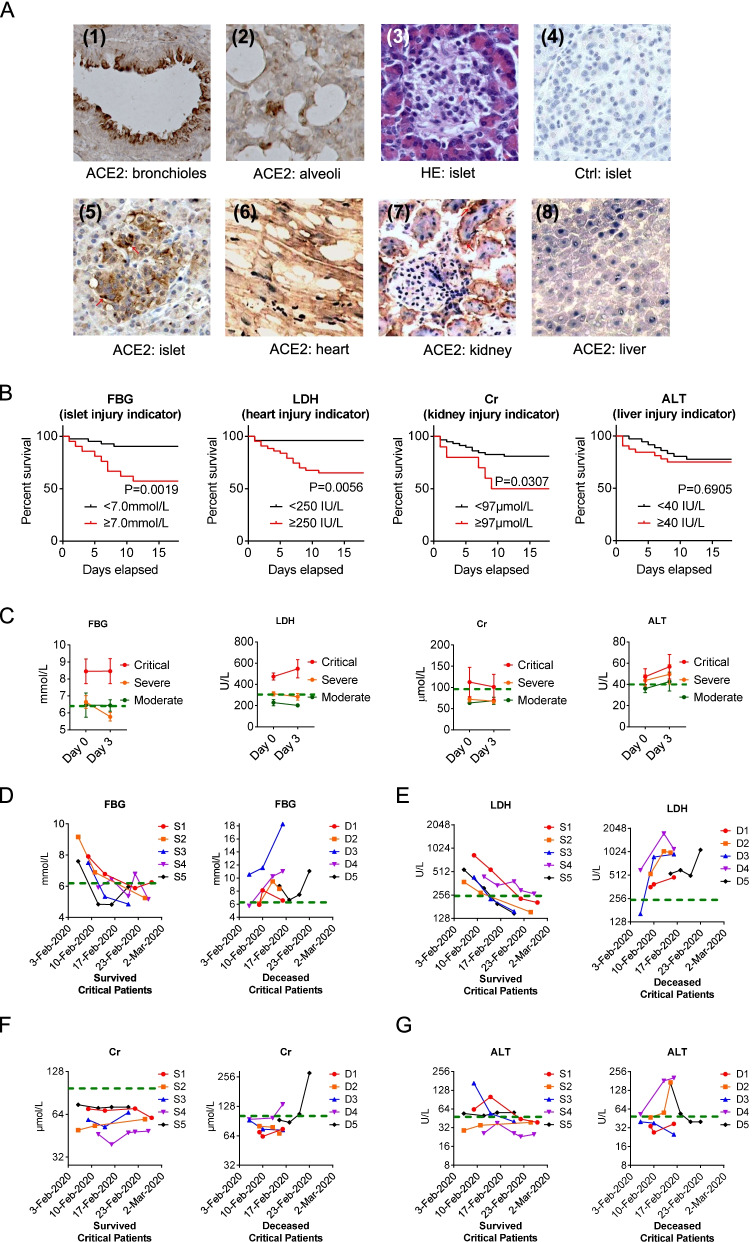
Profile of ACE2 multi-organ expression and corresponding multi-organ injury. **A** Immunohistochemically pattern of ACE2 (SARS-CoV-2 receptor) protein expression in different organs. (1, 2) Lung: high expression of ACE2 was found in (1) bronchiolar epithelial cells and (2) alveolar epithelial cells. (3–5) Pancreas: serial sections including (3) Hematoxylin-Eosin (HE) stain showing the exocrine tissue of pancreas around and a pancreatic islet in the middle, (4) negative immunostaining control showing no non-specific staining, (5) Islet: ACE2 was strongly positive in endocrine pancreas compared with exocrine tissue. (6) Heart: ACE2 was present in the myocytes, myocardium, border zone, endothelium of small-to-large arteries as well as sporadically within the smooth muscle of these vessels. (7) Kidney: ACE2 was very weakly present in glomerular visceral and parietal epithelium, but strongly present in the brush border and cytoplasm of proximal tubular cells, and in the cytoplasm of distal tubules and collecting ducts. (8) Liver: ACE2 was weak in hepatocytes and other cells including the endothelium of sinusoids. **B** Kaplan–Meier survival curves for in-hospital death rate of patients with COVID-19 subgroup by multi-organ injury indicators including: FBG ≥ 7.0 mmol/L for the pancreatic islet; LDH ≥ 250 IU/L for the heart; Cr ≥ 97 μmol/L for the kidney and ALT ≥40 IU/L for the liver. Log-rank test. **C** At the time of admission (Day 0) and after 3 days of hospitalization (Day 3), levels of FBG, LDH, Cr and ALT among *Critical*, *Severe* and *Moderate* patients with COVID-19 (mean ± s.e.m., *Moderate*, n = 23; *Severe*, n = 20; and *Critical*, *n* = 26). **D-G** Longitudinal observation during admission and subsequent treatment, levels of **D** FBG, **E** LDH, **F** Cr, and **G** ALT in five survived (S1-S5) *Critical* patients (Left) and in five deceased (D1-D5) *Critical* patients (Right)

To investigate multi-organ injury caused by the virus, the SARS-CoV-2 receptor, ACE2 protein expression pattern was analyzed in tissues, including the lung, heart, kidney, and liver which was obtained from the organ donor. In the lung, ACE2 immunostaining was abundant in epithelial cells of bronchioles and pulmonary alveoli. In the heart, it was highly expressed in the myocardium. In the kidney, ACE2 was strong in glomerular parietal epithelial cells and weak in the visceral epithelial cells. ACE2 expression was weak in the liver (Fig. 1A).

ACE2 was intensely stained in the pancreatic endocrine islets, but very weakly stained in pancreatic exocrine tissues (Fig. 1A).

Elevated fasting blood glucose (FBG) (≥ 7.0 mmol/L) defined as CRD can predict death (Fig. 1B). Although pancreatic islet dysfunction may not be the only reason of hyperglycemia in these patients. LDH, creatinine (Cr), and ALT were used as the relative function of multiple organs (i.e., the heart, kidney, and liver function, respectively). Kaplan-Meier analysis revealed a significantly higher in-hospital mortality rate for patients with elevated indicators for relative multi-organ injury. In parallel with the expression profiles of ACE2 in different organs, indicators of the relative dysfunction of multiple organs (LDH ≥ 250 IU/L for the heart, Cr ≥ 97 μmol/L for the kidney and ALT ≥40 IU/L for the liver, defended by previous report (Guan et al. 2020)) can predict death, except the liver function. (Fig. 1B).

### Longitudinal changes of multi-organs injury indicators

FBG and LDH levels were much higher above the upper limit of normal value at the time of admission (Day 0) and at the time after 3 days of hospitalization (Day 3) in the *Critical* group, while FBG and LDH levels did not increase remarkably in *Severe* and *Moderate* groups. However, Cr and ALT levels were not remarkably different among *Moderate*, *Severe* and *Critical* groups (Fig. 1C).

To further find the predictive values of these four indicators, a longitudinal case-control investigation was conducted in *Critical* patients, considering that the deaths happened only in *Critical* patients in this cohort. The *Critical* patients who were measured once every 3 days and at least three times in a row were analyzed. Although FBG and LDH levels were much higher above the upper limit of normal value at the time of admission, they dramatically decreased to the normal range during subsequent treatment in *Critical* patients who survived. However, of the patients who died, increases of FBG and LDH levels continued (Fig. 1D-E). These differences between survived and deceased patients were not found in Cr and ALT levels (Fig. 1F-G).

### Blood glucose is a representative indicator for mortality

**Fig. 2:**
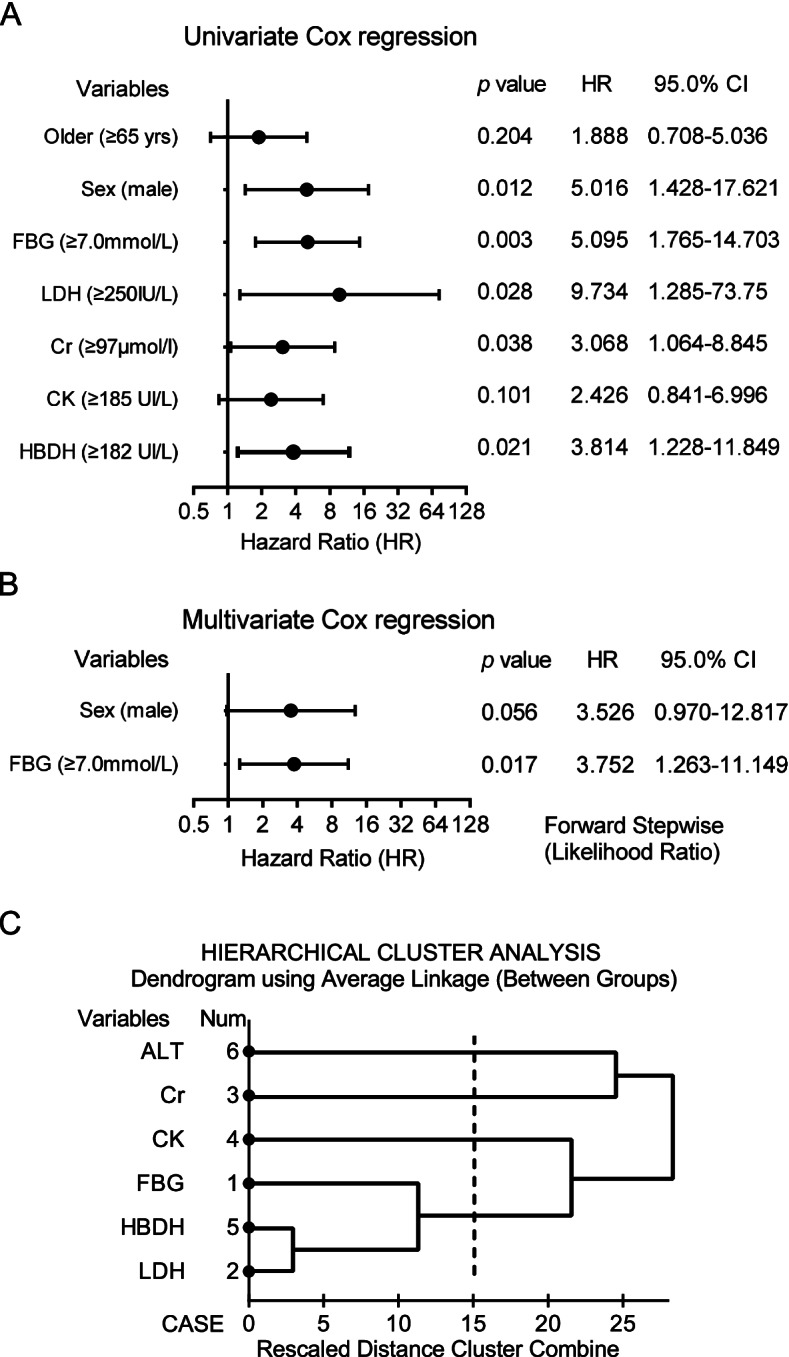
Effects of multi-organ injury indicators on the risk of in-hospital death in patients with COVID-19. **A** Univariable Cox regression indicated effects of multi-organ injury indicators on predicting in-hospital death. **B** Multivariable Cox regression indicated effects of independent multi-organ injury indicators on predicting in-hospital death. **C** Hierarchical cluster analysis of six indicators indicated these indicators were interdependent to each other

Using the univariable Cox proportional hazards model, male sex (HR = 5.02, 95% CI 1.43–17.62), elevated FBG (≥ 7.0 mmol/L) defined as CRD (HR = 5.09, 95% CI 1.76–14.70), LDH (HR = 9.73, 95% CI 1.28–73.7) and HBDH (HR = 3.81, 95% CI 1.23–11.85) were predictors for death (Fig. 2A). In the multivariable model, CRD remained the independent predictor for death (HR = 3.75, 95% CI 1.26–11.15) (Fig. 2B).

Cluster analysis of six indicators was performed to find if there were interdependent. Heart injury indicated by LDH/HBDH and FBG was in a cluster solution according to the Hierarchical cluster analysis (Fig. 2C). At the time of admission (Day 0), significant proximities or interdependent of LDH (Day 0) with HBDH (Day 0) (*r* = 0.73, *P* < 0.01) and CK (Day 0) with HBDH (Day 0) (*r* = 0.49, *P* < 0.01) were present. These results suggested that some definite indicators for multi-organ failure were independent. At the time after 3 days of hospitalization (Day 3), more significant proximities among Variables (Day 3), especially FBG (Day 3) with other Variables (Day 3) were found. These suggested that multi-organ injury might present at the same time caused by the virus infection. Most interestingly, significant proximities or interdependent of FBG (Day 0) with other Variables (Day 3) including LDH (Day 3) (*r* = 0.43, *P* < 0.01), HDDH (Day 3) (*r* = 0.43, *P* < 0.01), Cr (Day 3) (*r* = 0.50, *P* < 0.01) were present. Therefore, abnormal glucose homeostasis occurred earlier than other indicators for poor outcomes (Table 3).

**Table 3:** Proximity matrix of six multi-organ injury indicators by Hierarchical cluster analysis

	FBG	LDH	Cr	CK	HDDH	ALT
**FBG**	1					
**LDH**	**0.433	1				
**Cr**	**0.498	0.265	1			
**CK**	0.025	*0.328	0.113	1		
**HDDH**	**0.428	**0.881	0.128	**0.385	1	
**ALT**	0.099	**0.441	0.258	0.017	0.246	1

*FBG* Fasting Blood Glucose, *LDH* Lactate dehydrogenase, *Cr* Creatinine, *CK* Creatine kinase, *HDDH* Hydroxybutyrate dehydrogenase, *ALT* Alanine aminotransferase

** *p* < 0.01; * *P* < 0.05. Hierarchical cluster analysis, Pearson correlation test (2-tailed)

## Discussion

Except pneumonia, the multi-organ nature of SARS-CoV-2 infection has been demonstrated in a latest autopsy studies (Yao et al. 2020). Moreover, an autopsy study on 3 cases of SARS indicate that some lesions observed in heart, kidney, pancreas may have existed before the hospitalization (Lang et al. 2003). We found that the SARS-CoV-2 receptor, ACE2 is highly expressed in multiple organs including the lung, the heart, the kidney and the pancreatic islet. In this study, we identified early multi-organ injury indicators for predicting severity and mortality in patients with COVID-19. Our results suggest that blood glucose is a representative of the clustered multi-organ injury indicators and earlier predictor for poor outcomes and death in these patients.

Acute heart injury (AHI) is common in patients with viral infection, such as adenovirus, herpesvirus and enterovirus (Pollack et al. 2015). Since carditis associated with coronavirus infection was first reported in 1980 (Riski et al. 1980), growing evidence shows that coronavirus is also a pathogen for AHI that should not be ignored. Early in 2006, we reported that SARS-CoV may cause AHI and elevated LDH is an independent predictor for in-hospital death in SARS patients (Yang et al. 2010). A report from Saudi Arabia indicated that Middle East Respiratory Syndrome coronavirus (MERS-CoV) could cause acute myocarditis and acute-onset heart failure (Alhogbani 2016). The latest case report described cardiac involvement in a patient with COVID-19 (Inciardi et al. 2020). Two myocardial enzymes, LDH and HBDH exist in myocardia and release into blood flow once the myocardial injury happens. In this study, we found that both high LDH and HBDH on admission were predictor for severity and in-hospital death.

Acute kidney injury (AKI), is also common in patients with virus infection, such as Parvovirus B19, Hanta, Ebola, and Dengue virus infection (Prasad et al. 2019). Coronavirus associated AKI was reported in patients with MDRS (Mackay and Arden 2015). In the previous report, we found that SARS-CoV may cause AKI and elevated Cr is an independent predictor for in-hospital death in SARS patients (Yang et al. 2010). In this study, we found that elevation of Cr was present in *Critical* and deceased patients. High Cr on admission were predictor for severity and in-hospital death in COVID-19 patients.

Acute liver injury (ALI) was reported in SARS patients, which was manifested with mild elevation of ALT/AST levels. Decreased serum albumin level was present in some patients. However, ALT and AST was not an independent predictor of poor outcome (Wu et al. 2004). A number of studies have shown that elevated liver enzymes and decreased albumin levels were present in patients with MERS (Xu et al. 2020b). Case reports suggested that patients with severe COVID-19 seem to have higher rates of liver dysfunction (Cui et al. 2020). However, few studies have shown the liver enzymes were independent predictor for poor outcome and mortality in COVID-19 patients. In this study, although mild decrease of albumin was found in *Critical* and deceased patients, significant differences of liver enzymes were not found between survivors and deceased patients in this cohort. We propose that liver injury may not be a direct damage caused by the virus, because we found that expression of the SARS-CoV-2 receptor, ACE2 in the liver is low.

Acute pancreatic islet injury (AisI) caused by virus infection has scarcely been reported. Subacute islet injury caused by virus infection has been widely reported in type 1 diabetes, which is an autoimmune disease characterized by a long-term loss of pancreatic islet β-cells. However, serological evidence of infection and isolation of viruses from the pancreas have been reported in a few cases of recently diagnosed acute diabetes (Jaeckel et al. 2002). A case-control study indicated that high FBG is an independent predictor for severity of H1N1 pneumonia. In this study, FBG was remarkably increased in patients with H1N1 pneumonia than in patients with non-H1N1 pneumonia (8.3 vs. 6.2 mmol/L). Moreover, compensative rise in insulin, in corresponding with high FBG was not found; instated, islet β-cells function indicated by HOMA-β index is relatively lower (Wang et al. 2011).

It is reasonable to think that FBG elevation maybe caused by “Stress hyperglycemia” or insulin resistance during acute illness in patients with COVID-19. However, severe hyperglycemia is related to both insulin secretion and insulin resistance. Insulin resistance caused by metabolic, hormonal, and cytokine changes associated with the illness demands a corresponding rise in insulin output in order to maintain normal glycemia. Only when the compensation is lost will blood glucose increase significantly (Gupta et al. 2012; Dungan et al. 2009). Therefore, relative dysfunction of pancreatic islet insulin secretion caused by the virus infection maybe the main reason of severe hyperglycemia in patients with COVID-19.

There are several limitations in our study. First, due to the critical condition of the disease in early 2020, laboratory data at some points is missing. Second, we only assessed multi-organ injury from the perspective of some most accessible biochemical parameters. It would have produced better results if some more specific indicators such as echocardiography, glomerular filtration rate and serum insulin levels were also measured in this study, and if we could assess some organ injury-specific mortality. Last but not least, interpretation might be limited by the sample size of the study. However, by including all patients of department of critical care medicine in the designated hospitals, we believe our study population of COVID-19 is a representative of patients treated in Wuhan.

In summary, except pneumonia, multi-organ injury including the heart, kidney, and possibly pancreatic islet injuries was already occurred at an early stage and thereby increased the risk of mortality later in patients with COVID-19. These multi-organ injury indicators including LDH, HBDH, CK, Cr and FBG were associated with higher odds of primary outcomes and death. However, these indicators are interdependent, indicating that the multi-organ damage happened at the same time. Among them, FBG is a representative of the clustered multi-organ injury indicators for predicting mortality of COVID-19. As it is easy to perform for clinical practices and self-monitoring, FBG testing will be much helpful for predicting critical condition to facilitate appropriate intensive care.

## Data Availability

JKY is the guarantor of this work and, as such, the data that support the findings of this study are available from him upon reasonable request.
